# Therapeutic and Preventive Effects of *Olea europaea* Extract on Indomethacin-Induced Small Intestinal Injury Model in Rats

**DOI:** 10.1155/2020/6669813

**Published:** 2020-12-23

**Authors:** Fatemeh Sadat Mahdavi, Parham Mardi, Seyed Saeed Mahdavi, Mohammad Kamalinejad, Seyed Ali Hashemi, Zohreh Khodaii, Mahboobeh Mehrabani-Natanzi

**Affiliations:** ^1^Student Research Committee, Alborz University of Medical Sciences, Karaj, Iran; ^2^Faculty of Medicine, University of Debrecen, Debrecen, Hungary; ^3^Department of Pharmacognosy, School of Pharmacy, Shahid Beheshti University of Medical Sciences, Tehran, Iran; ^4^Pathology Department, Faculty of Medicine, Alborz University of Medical Sciences, Karaj, Iran; ^5^Dietary Supplements and Probiotic Research Center, Alborz University of Medical Sciences, Karaj, Iran; ^6^Evidence-Based Phytotherapy and Complementary Medicine Research Center, Alborz University of Medical Sciences, Karaj, Iran

## Abstract

**Background:**

*Olea europaea* (known as olive fruit) has anti-inflammatory and antioxidant activities and many potential health benefits including gastric inflammation reduction has been shown previously. This study aimed to investigate the preventive and therapeutic effects of *O. europaea* extract on the early histological changes in indomethacin-induced small intestinal injury model with the plasma D-lactate concentration being measured as a tool for determination of intestinal permeability.

**Methods:**

In this experimental study, two separate protective and therapeutic protocols were designed. In both experiments, male Wistar rats were randomly divided into 4 groups and either pretreated with 0, 100, 200, or 400 mg/kg/day of *O. europaea* extract or received the treatment after administration of indomethacin. Their small intestines were examined to compare the histological changes. The intestinal injury severity was evaluated according to the presence of eosinophils, plasma cell infiltration, edema, congestion, and hyperplasia of the crypt using a histological scoring system. Also, measured were the presence of neutrophils, decreased villus length-to-crypt depth ratio, and destructed villus architecture. The plasma concentration of D-lactate was measured as well.

**Results:**

The therapeutic use of *O. europaea* decreased the eosinophil, edema, congestion, and crypt hyperplasia scores compared to the control group. Although no significant difference was seen between groups of the preventive experiment in plasma cell infiltration score, villus length-to-crypt depth ratio, neutrophil infiltration, and percentage of destructed villus architecture, treatment with *O. europaea* caused a reduction in edema, eosinophil, congestion, and crypt hyperplasia score. In both experiments, no significant difference was seen between groups for villus length-to-crypt depth ratio, neutrophil infiltration, and percentage of destructed villus architecture. Plasma D-lactate concentration was decreased in all *O. europaea*-treated groups compared to the control group in the therapeutic and preventive experiments (*p* < 0.01, one-way ANOVA followed by the Dunnett test).

**Conclusion:**

*O. europaea* extract can be used to decrease some side effects of indomethacin on intestinal tissue and enhances the gastrointestinal function. *O. europaea* extract could be considered as a potential herbal supplement in the treatment of intestinal morphological injuries.

## 1. Background

Nonsteroidal anti-inflammatory drugs (NSAIDs) are a group of anti-inflammatory drugs used to decrease pain and to treat immunological and rheumatological disorders [1, 2]. Peptic ulcer disease and intestinal inflammation are well-recognized complications of NSAID use [3, 4]. Indomethacin is an NSAID drug commonly used to reduce fever, pain, and stiffness and to treat gout and arthritis. Indomethacin prevents the synthesis of prostaglandins that prevent inflammation of the intestine and also disrupts blood circulation in the submucosa, causing dysregulation in mucosal cell growth that leads to hyperplasia [5, 6].

Structural and functional changes of intestinal mucosa can cause barrier dysfunction, which may lead to an increase in the permeability of the intestine [7]. Among all NSAIDs, indomethacin induces highest permeability changes. Increased intestinal permeability causes the passage of lipopolysaccharide and bacterial toxins into the bloodstream and, therefore, becomes a factor for several local and systemic inflammations [8, 9].

Antirheumatic, anti-inflammatory, antipyretic, vasodilatory, hypotensive, diuretic, and hypoglycemic effects of the olive fruit (*Olea europaea* L.) have been studied and described. These effects are attributed to monounsaturated fatty acids, aliphatic and triterpene alcohols, sterols, hydrocarbons, and several antioxidants that are present in this fruit. Potential health benefits of *O. europaea* against chronic diseases can be explained by its antioxidant activity and prevention of the harmful effects of free radicals. The antioxidant capacity of *O. europaea* is mainly due to oleuropein [10–12]. Prior studies have shown that *O. europaea* could reduce stomach inflammation caused by indomethacin [13, 14]. This study aims to investigate the preventive and therapeutic effects of *O. europaea* extract on the first histological changes in indomethacin-induced small intestinal injury models, and the plasma D-lactate concentration is measured as a tool for determination of intestinal permeability.

## 2. Methods

### 2.1. Plant Collection and Preparation of Extract

Green olive fruits (*Olea europaea*), collected at the end of the summer, were obtained from the Rudbar region, Gilan, Iran, and the fruits were scientifically authenticated by qualified field botanist (Prof. Maryam Ahvazi) at Medicinal Plants Research Center, Institute of Medicinal Plants, ACECR, Karaj, Iran. The voucher samples were deposited in the Herbarium and Raw Drug Repository (Alborz University of Medical Sciences). After cleaning the olive fruits, they were dried by exposure to air at room temperature and away from direct sunlight. The fruits were crushed and extracted with 80% ethanol (4 times per day, 20 cc solvent each time, for 25 days) in a percolator [15]. After extraction, the solvents were evaporated by a rotatory evaporator. The ethanol extract was stored in closed and dark containers at −20°C until used in the experiment.

### 2.2. Animals

In this experimental study, a total of 54 male Sprague-Dawley rats weighing 200–250 g at six weeks were obtained from Royan Research Institute (Tehran, Iran). The rats were kept in the animal facility at the Alborz University of Medical Sciences for one-week before starting experimentation at a temperature of 22 ± 2°C with a relative humidity of 50% and a 12 : 12-hour light-dark cycle. Additionally, they had free access to a standard diet and water. The study was carried out following the institutional and international guidelines after approval by the Ethical Committee of Alborz University of Medical Sciences (Abzums.Rec.1397.024).

## 3. Experimental Protocol

### 3.1. Therapeutic Experimental Design

#### 3.1.1. Induction of Small Intestinal Injury

A total of 30 male Sprague-Dawley rats were used to evaluate the curative effect of the *O. europaea* extract on indomethacin-induced small intestinal injury model. Indomethacin was administrated in 27 rats to induce histological injury to the small intestine. Three rats were administered by oral gavage with distilled water for three days as a healthy control group. Twenty-seven male Wistar rats were orally administered indomethacin 15 mg/kg *q* 12 hours for three days [16] (Sigma-Aldrich, 17378-5G, Germany). On day 4, randomly, 3 of the affected rats (3-day control group) and the healthy control group were euthanized, and their small intestines were removed. The small intestinal injury was investigated and compared.

#### 3.1.2. Administration of the *O. europaea* Extract

After inducing injury with indomethacin, the remaining rats were randomly divided into four groups (6 rats each). From day 4, animals in the 10-day control group received no extract medication, but they were given an oral gavage of distilled water (0.5 cc) daily for seven days. Each of the other three treatment groups received either 100, 200, or 400 mg/kg of *O. europaea* extract daily from day 4 to day 10 [17].

After ten days, all rats were humanely dissected, blood samples were collected from the rat heart and collected into vacuum tubes which contained sodium citrate as anticoagulant, and the small intestine was immediately removed for histological studies.

### 3.2. Preventive Experimental Design

For evaluating the preventive effect of *O. europaea* extract, a separate group of 24 male Sprague-Dawley rats were used, and they were randomly divided into four groups. The rats were initially pretreated orally with distilled water (0.5 cc) in the prevention control group, and either 100, 200, or 400 mg/kg of *O. europaea* extract daily in the three prevention groups for four days. On day 5, indomethacin 15 mg/kg *q* 12 hours started to be administrated plus either distilled water (0.5 cc) in the prevention control group, or plus 100, 200, or 400 mg/kg of *O. europaea* extract in the prevention groups for three days. After seven days, all rats were euthanized, and blood and intestinal tissue samples were collected.

**Figure 1:**
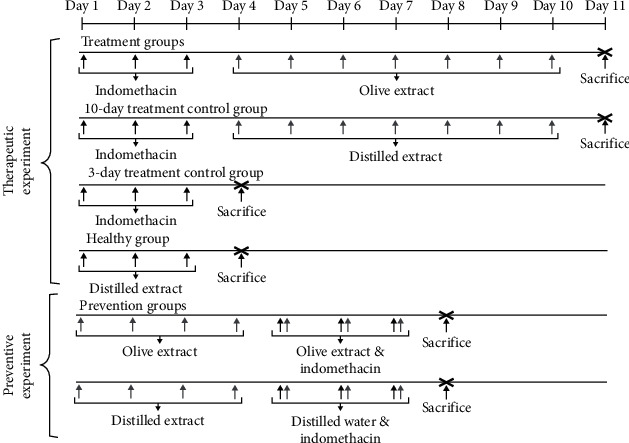
Therapeutic and preventive experimental designs.

Both therapeutic and preventive experimental protocols are summarized in Figure 1.

## 4. Histological Studies

At the end of the experiment, the rats were euthanized by intraperitoneal injection of sodium pentobarbitone (concentration of 60 mg/ml) at a dose of 180 mg/kg. Thereafter, the confirmation of euthanasia was made by absence of the tail pinch reflex. After gathering the blood samples, rats were sacrificed by cervical dislocation. The abdomen was opened, and the small intestine excised. Histological observations were carried out on jejunal segments from rats of each group. Jejunum segments were immediately injected with 10% formalin and left in the same fixative solution for 30 minutes. Then, the sections were dissected along the antimesenteric border and cleaned of fecal content. Eventually, they were fixed in a 10% formalin for 24 hours. Four sections were randomly chosen from each of the jejunum segments. Hematoxylin and eosin (H&E) staining was performed in routinely processed paraffin-embedded sections. Histological alterations were observed by two independent pathologists without previous knowledge of samples' study group origin [16, 18].

**Figure 2:**
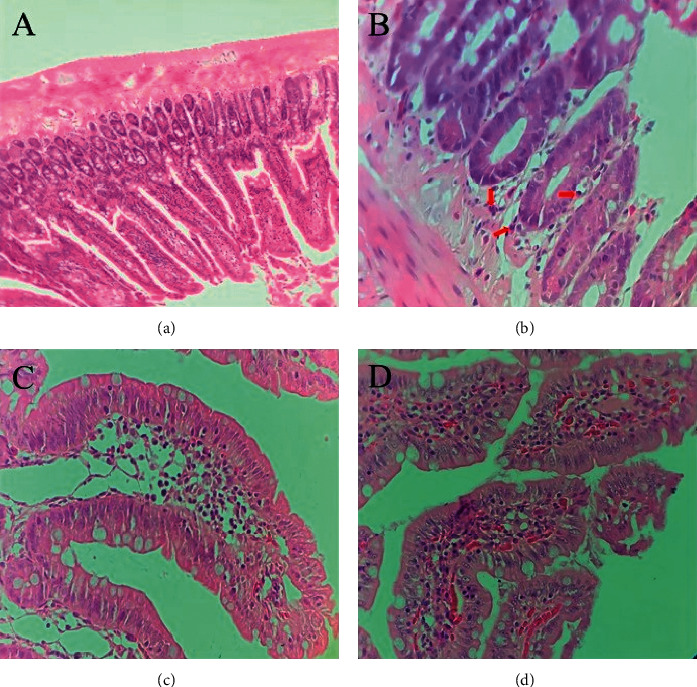
Histological alterations in the small intestine. Images show the H&E-stained sections from jejunum. (a) Normal intestinal wall structure without histological abnormalities in rats of the healthy group (magnification ×10). (b) Arrows show eosinophils in the epithelium of the intestine in rats of the control group (magnification ×40). (c) A grade 3 villus edema and (d) a grade 3 congestion, observed in rats of the control group (magnification ×40).

The histological sections were evaluated using standard light microscopy, and the intestinal injury severity was evaluated by assessing the presence of eosinophils, plasma cell infiltration, neutrophils, edema, congestion, hyperplasia of the crypt, decreased villus length-to-crypt depth ratio, and destructed villus architecture (Figure 2).

The numbers of eosinophils in four different microscopic fields (40×) were counted, and the mean number was reported. Then, it was categorized according to eosinophil level into mild (0 to 10 eosinophils), moderate (11 to 20 eosinophils), or severe (more than 20 eosinophils). The severity of plasma cell infiltration in the lamina propria, edema, and congestion was reported in three grades of mild, moderate, and severe as well.

Hyperplasia of the crypt is defined as observing more than one mitosis in a single crypt. Reporting hyperplasia in up to 25% of the crypts visible in a low power microscopic field is classified as mild, 25% to 50% as moderate, and more than 50% as severe crypt hyperplasia. The presence of neutrophils was being investigated, and even a single cell considered important enough to be reported.

For villus length-to-crypt depth ratio measurement, three villi and three intestinal crypts per slide were studied, and only those regions of the intestinal sections presenting proper morphology were used. Histomorphometry measurements were carried out under low microscopic power. The height of each villus was defined as the length from top of the villus to the crypt transition, and the crypt depth as the length from the villus-crypt junction to the crypt base. Normal or destructed villus architectures were reported according to the pathologists' subjective opinion. In case of a difference of opinion between the two pathologists, a third opinion from another expert was requested.

## 5. D-Lactate Measurement

Heart blood samples were collected just after the rats were euthanized, for serum D-lactate level assessment. The plasma from systemic blood samples was obtained and subjected to deproteination and neutralization processes by acid/base precipitation using perchloric acid and potassium hydroxide. The protein-free plasma was then assayed for D(-)-lactate concentration (mmol/L) by the enzymatic-spectrophotometric method with minor modifications [19].

## 6. Statistical Analysis

Using SPSS 22.0 software (SPSS Inc., Chicago, IL, USA), D-lactate levels were compared within groups by one-way ANOVA followed by the Dunnett test. One-way ANOVA and chi-square are used to evaluate the difference between groups in terms of the crypt-to-villus ratio and architecture disturbance, respectively. The Mann–Whitney *U* test was used for plasma cell infiltration, edema, eosinophil, congestion, and hyperplasia. A *p* value of <0.05 was considered to be statistically significant, and all data were expressed as mean ± standard deviation (SD) in each group.

## 7. Results

**Figure 3:**
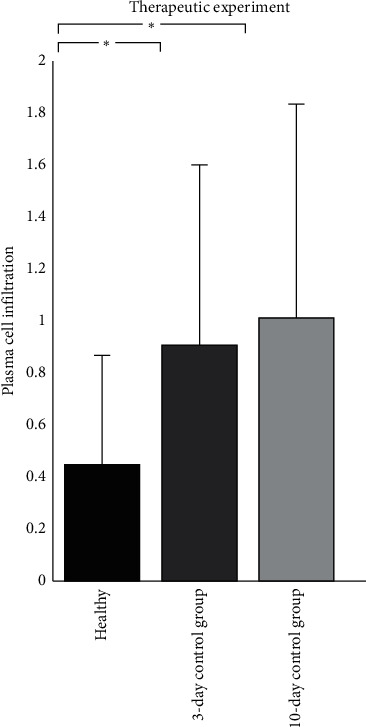
Plasma cell infiltration scores were compared to ensure the induction of indomethacin-induced small intestinal injury in rats. Data are expressed as mean ± standard deviation. The plasma cell infiltration score was significantly lower in the healthy group compared to the three-day control or ten-day control group (^*∗*^*p* value <0.05).

To ensure the induction of indomethacin-induced intestinal injury in rats, the plasma cell infiltration score was compared between the healthy group and the three-day control groups. The plasma cell infiltration score was significantly lower in the healthy group compared to the three-day control or ten-day control group (Figure 3).

The therapeutic effects of *O. europaea* against indomethacin-induced intestinal injury were evaluated. *O. europaea*, at a dose of 100 mg/kg, decreased the eosinophil, edema, and crypt hyperplasia scores compared to the control group. 200 mg/kg of *O. europaea* caused a reduction in eosinophil, hyperplasia, plasma cell infiltration, and congestion scores. Eosinophil, crypt hyperplasia, and plasma cell infiltration scores were eliminated in the 400 mg/kg dose group in comparison with the control group (*p* < 0.05, Mann–Whitney). No significant difference in groups was seen between villus length-to-crypt depth ratio, neutrophil infiltration, and percentage of destructed villus architecture (*p* > 0.05, one-way ANOVA, and chi-square, respectively).

**Figure 4:**
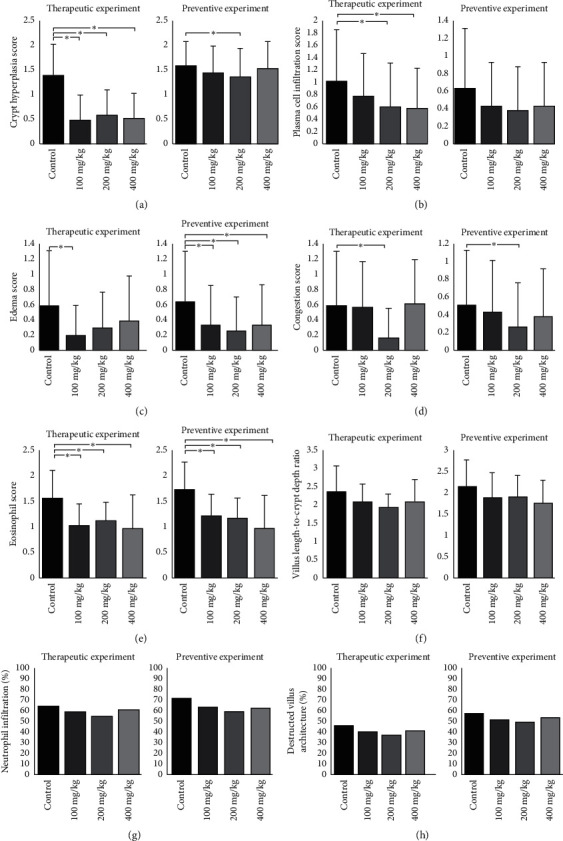
Histological changes in the small intestine. (a) Crypt hyperplasia score was decreased in all doses of *O. europaea* compared to the control group in the therapeutic experiment. However, only *O. europaea* at a dosage of 200 mg/kg was effective in the preventive experiment. (b) Plasma cell infiltration scores were lower in the dosage of 200 and 400 mg/kg of *O. europaea* in the therapeutic experiment. Although, no significant difference in groups of preventive experiment was seen. (c) Edema score was decreased in the dosage of 100 mg/kg of *O. europaea* in the therapeutic experiment and all doses in the preventive experiment. (d) Congestion scores were lower in a dosage of 200 mg/kg of *O. europaea* in both experiments. (e) All groups treated with *O. europaea* in therapeutic and preventive experiments showed a significant reduction in eosinophil scores. (f) Villus length-to-crypt depth ratio was not changed between groups of both experiments. Data in sections A to F are expressed as mean ± standard deviation. (g) Presence of neutrophils is expressed as percent indicating the percentage of the study population that was found to show at least one neutrophil during our investigations. We did not find any statistically significant difference anywhere in our experiments. (h) Percentage of destructed villus architecture was not different between any of the study groups. ^*∗*^*p* value <0.05.

To assess the preventive effects of *O. europaea* against indomethacin-induced intestinal injury, *O. europaea* was administered by oral gavage for four days before oral administration of indomethacin. Intestinal biopsy revealed lower edema and eosinophil score in all prevention groups compared to the control group. Also, *O. europaea*, at a dose of 200 mg/kg, decreased congestion and crypt hyperplasia score (*p* < 0.05, Mann–Whitney). No significant difference was seen between groups for plasma cell infiltration score, villus length-to-crypt depth ratio, neutrophil infiltration, and percentage of destructed villus architecture (*p* > 0.05, Mann–Whitney, one-way ANOVA, and chi-square, respectively) (Figure 4).

**Figure 5:**
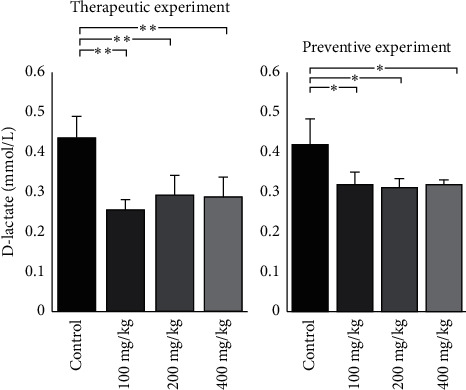
The D-lactate concentration (mmol/L) was decreased in all *O. europaea*-treated groups in comparison to the control group of the therapeutic experiment (*p* < 0.01). Also, in the preventive experiment, D-lactate concentration was significantly lower in all *O. europaea*-treated groups compared to the control group (*p* < 0.05). All data are expressed as mean ± standard deviation. ^*∗*^*p* value <0.05 and ^*∗∗*^*p* value<0.01.

Histological studies were followed by plasma D-lactate concentration analysis in both preventive and therapeutic experiments. In the therapeutic experiment, the D-lactate concentration was decreased in all *O. europaea*-treated groups in comparison with the control group (*p* < 0.05, one-way ANOVA followed by the Dunnett test). Also, in the preventive experiment, D-lactate concentration was significantly lower in all *O. europaea*-treated groups compared to the control group (*p* < 0.05, one-way ANOVA followed by the Dunnett test) (Figure 5).

## 8. Discussion

NSAIDs often cause mucosal lesions in the small intestine in humans [20]. Researchers are diverting attention from using aminosalicylates and glucocorticoids for the treatment of NSAID-induced intestinal inflammation to new therapeutic strategies such as using antioxidants [21–25]. NSAID-induced lipid peroxidation and oxidative stress cause ulcers in the gastrointestinal mucosa [26]. Some reports have indicated that oral administration of NSAIDs cause gastrointestinal oxidative injury through increased lactate dehydrogenase (LDH) leakage, mucosal lipid peroxidation (MDA), DNA damage, and decreased gastric mucus secretion in vivo. Thus, upregulation of antioxidant enzymes, such as glutathione peroxidase (GPx), glutathione reductase (GR), superoxide dismutase (SOD), and heme oxygenase-1 (HO-1), might be a major mechanism of action against oxidative stress-associated gastrointestinal ulcers [27–29].

Several studies have investigated the phenolic composition of the olive fruit. The phenolic compounds present in *Olea europaea* L., especially the oleuropein, are associated with antioxidant, antihypertensive, hypoglycemic, hypocholesterolemia, and cardioprotective activity [30, 31]. Oleuropein is a potent antioxidant with anti-inflammatory properties [32]. Prevention of free radical formation by oleuropein may be due to its ability to chelate metal ions, such as copper (Cu) and iron (Fe), which catalyze free radical generation reactions, as well as its ability to inhibit several inflammatory enzymes, such as lipoxygenases [33, 34].

Prior studies demonstrate that *O. europaea* L. extract has protective function in certain diseases [35, 36]. This study evaluates the preventive and therapeutic effects of *O. europaea* L. fruits on indomethacin-induced intestinal injury using histopathological studies and D-lactate level assessment.

Preventive administration of *O. europaea* L. at dose 200 mg/kg/day decreased edema, congestion, crypt hyperplasia, and eosinophil score in the histological evaluation and D-lactate level compared to the control group.

Dan et al. showed that the eosinophil number in the histological study is not only attributed to the severity of inflammation but can be used as an index to assess the treatment efficacy [37]. Crypt hyperplasia is characterized by an increased number of mitoses, due to the extension of the proliferative compartment from the crypt bases along the length of the crypt. Crypt hyperplasia is a valid and reliable measure of intestinal inflammation and its response to treatment effects of preclinical studies [38]. Our findings indicate that preventive administration of *O. europaea* in a dose of 200 mg/kg and therapeutic administration of the extract in all doses decrease crypt hyperplasia and eosinophil infiltration. Although a different eosinophil scoring system was used in our study, Ciobanu et al. have demonstrated that rifaximin decreases infiltrated eosinophils number, concluding that it is also efficient in preventing degenerative intestinal lesions induced by indomethacin [39].

It has been proposed that the infiltration of the bowel mucosa by neutrophils is an essential stage in NSAIDs' injuries. Similar to Ciobanu et al.'s study [39], in our study, assessment of neutrophil infiltration was not different between any of our preventive, therapeutic groups compared to the control group, suggesting that other mechanisms may also be involved. Watanabe et al. reported that lipopolysaccharides (LPS)/toll-like receptor 4 (TLR 4)/MyD88-dependent signaling pathway on macrophages plays an essential role in the activation of an inflammatory cascade induced by NSAIDs. In addition, a bias might be present in our scoring system, which would explain the results [40, 41].

Indomethacin not only causes vasoconstriction but increases the risk of kidney problems, ischemic heart disease, and heart failure [42–44]. In our study, edema was significantly decreased in all our preventive experimental groups compared to the control group, and there was a discrepancy in our therapeutic experimental findings, in terms of the edema score. The results of our congestion scores were also inconclusive. The discrepancy in our findings may be attributed to preventable but irreversible cardiac effects of indomethacin. Despite this, edema and congestion appear to be unreliable indicators of indomethacin-induced small intestinal injury [45, 46].

Crypt-to-villus ratio and villus architecture took longer to change. These two indices were not altered in both of our experiments. Due to our short experiment duration, a more extended follow-up period may be required to detect any differences [45, 47, 48].

In our study, unlike the preventive experiment, the therapeutic experiment showed a significant decrease in plasma cell infiltration as a factor indicative of inflammation. We suggest that different immunological responses may justify the contrast between our therapeutic and preventive experiments [49, 50].

Intestinal permeability is an essential factor when assessing the effects of NSAIDs on intestinal integrity [51, 52]. D-lactate is closely related to the intestinal integrity and is attributed to specific conditions such as leaky gut and NSAIDs induced intestinal injury. Prior studies indicate that D-lactate could be used as a major diagnostic index [53]. Confirming histological findings, the D-lactate level was significantly lower in all our *O. europaea*-treated groups in both therapeutic and preventive studies.

## 9. Conclusion

Overall, we conclude that, although *O. europaea* significantly reduced inflammation and injury caused by indomethacin, its preventive effects were less evident than its therapeutic effects on the indomethacin-induced intestinal injury. Our study revealed that the best dose was determined to be 200 mg/kg for both experiments.

## Data Availability

No data have been submitted to any open-access databases. All the data supporting the study are presented in the manuscript or available upon request.
